# Beyond BMI: An opinion on the clinical value of AI-powered CT body composition analysis

**DOI:** 10.17305/bb.2025.12774

**Published:** 2025-07-07

**Authors:** Matej Pekar, Marek Kantor, Jakub Balusik, Jan Hecko, Piotr Branny

**Affiliations:** 1 Complex Cardiovascular Center, Hospital AGEL Trinec-Podlesi, Trinec, Czech Republic; 2 Physiology, Faculty of Medicine, Masaryk University, Brno, Czech Republic; 3 Faculty of Electrical Engineering and Computer Science, VSB-Technical University of Ostrava, Ostrava, Czech Republic; 4 Cardiac Surgery, Faculty of Medicine, Palacky University, Olomouc, Czech Republic

**Keywords:** Body composition, artificial intelligence, AI, computed tomography, CT, sarcopenia, visceral adipose tissue, VAT

## Abstract

Body mass index (BMI) has long been used as a standard measure for assessing population-level health risks, but its clinical adequacy has increasingly been called into question. This opinion paper challenges the clinical adequacy of BMI and presents AI-enhanced CT body composition analysis as a superior alternative for individualized risk assessment. While BMI serves population-level screening, its inability to differentiate between tissue types leads to critical misclassifications, particularly for sarcopenic obesity. AI-powered analysis of CT imaging at the L3 vertebra level provides precise quantification of skeletal muscle index, visceral, and subcutaneous adipose tissues -metrics that consistently outperform BMI in predicting outcomes across oncology, cardiology, and critical care. Recent technological advances have transformed this approach: the “opportunistic” use of existing clinical CT scans eliminates radiation concerns, while AI automation has reduced analysis time from 15–20 minutes to mere seconds. These innovations effectively address previous implementation barriers and enable practical clinical application with minimal resource demands, creating opportunities for targeted interventions and personalized care pathways.

## Introduction

For decades, body mass index (BMI) has been the primary metric for assessing obesity in clinical practice. However, as our understanding of body composition and its relationship to health outcomes has advanced, the limitations of this simplistic measure have become increasingly evident [1, 2]. While BMI is useful for population-level screening, it fails to differentiate between fat and muscle mass, resulting in significant misclassification of individual risk profiles [3].

The rising prevalence of phenotypes such as sarcopenic obesity—characterized by reduced muscle mass coupled with increased adiposity—challenges traditional methods of body composition assessment. This scenario necessitates a paradigm shift in how we evaluate, categorize, and manage patients with diverse body composition profiles.

Our analysis presents compelling evidence that more sophisticated approaches to body composition assessment are not merely technical advancements but represent substantial improvements in patient care.

## Methods

This opinion article is informed by our clinical expertise in utilizing AI-powered computed tomography (CT) for body composition analysis, supplemented by a comprehensive yet non-systematic review of relevant literature. We conducted searches in the PubMed, Scopus, and Google Scholar databases from inception through January 2025, employing key terms such as “body composition”, “computed tomography”, “artificial intelligence”, “sarcopenia”, “visceral adipose tissue”, “BMI limitations”, and their combinations.

We prioritized peer-reviewed articles published between 2020 and 2025 that demonstrated clinical applications of CT-derived body composition analysis across medical specialties, validation studies of AI segmentation tools, comparative effectiveness studies between BMI and CT-based metrics, and consensus statements from relevant professional societies. Seminal older studies that established foundational concepts were also included regardless of publication date. We excluded studies focused solely on animal models, pediatric populations (due to differing reference standards), purely methodological papers lacking clinical applications, and studies without full-text availability in English.

Articles were selected based on their relevance to our clinical perspective and direct experience with AI-powered body composition assessment in cardiovascular patients. As an opinion article, our methodology integrates expert clinical insights with supporting literature rather than adhering to formal systematic review protocols. This approach allows us to convey our informed clinical opinion while providing appropriate scientific context through contemporary evidence.

### The growing challenge of body composition assessment

The landscape of obesity has changed significantly in recent decades. We now recognize that body composition, rather than simple weight metrics, plays a crucial role in determining health outcomes [2]. Traditional anthropometric measurements—including BMI, waist circumference, and waist-to-hip ratio—while accessible and widely used, provide an oversimplified view of an individual’s metabolic health and risk profile.

This perspective aligns with the consensus statement from the European Society for Clinical Nutrition and Metabolism (ESPEN) and the European Association for the Study of Obesity (EASO), which emphasizes the importance of recognizing sarcopenic obesity as a distinct clinical condition, characterized by the bidirectional pathogenic interactions between adiposity and muscle loss [4].

Multiple recent reviews demonstrate that CT-derived body composition measures often outperform BMI in predicting patient outcomes. A systematic review indicated that CT analysis detected sarcopenia 27%–67% more frequently than BMI-based screening [5]. In the field of oncology, CT-based metrics have shown greater prognostic value than BMI: Caan et al. [6] reported that low muscle mass and high adiposity on CT were associated with worse survival outcomes in breast cancer patients, whereas BMI alone failed to capture this risk.

In clinical practice, we frequently encounter patients who defy traditional categorizations. For instance, the paradox of metabolically healthy obesity (MHO) and the hidden risks associated with normal-weight central adiposity underscore a crucial reality: our conventional tools for assessing body composition often overlook critical information that could enhance clinical decision-making. The increasing prevalence of sarcopenic obesity—especially among aging populations—further complicates this issue, as it signifies a unique risk profile that traditional metrics fail to encompass [7].

### Advanced imaging and tissue quantification

Recent technological advancements have opened new avenues for detailed body composition assessment. CT imaging, particularly at the third lumbar vertebra (L3) level, has emerged as a powerful tool for precise tissue quantification [8]. This method enables accurate measurement of the skeletal muscle index (SMI), with established cutoff values for sarcopenia set at <38.9 cm^2^/m^2^ for women and <55.4 cm^2^/m^2^ for men [9–11]. It also facilitates the assessment of visceral adipose tissue (VAT) and subcutaneous adipose tissue (SAT), offering unprecedented insights into body composition profiles.

It is essential to define these key metrics clearly. The SMI refers to the cross-sectional area of skeletal muscle at L3, normalized to height squared (cm^2^/m^2^). Low SMI is associated with frailty, chemotherapy toxicity, surgical complications, and increased mortality. VAT refers to fat located within the abdominal cavity surrounding organs. While both VAT cross-sectional area (cm^2^) and tissue density (Hounsfield Units) are routinely measured, VAT density is the primary parameter used for metabolic risk assessment and clinical interpretation in our analysis. Higher VAT density values indicate increased tissue fibrosis and inflammation, reflecting more pronounced metabolic dysfunction. Based on our previous research employing maximization of log-rank statistics, we established gender-specific cutoff values for high-risk classification: VAT density >−93.27 HU for men and >−95.02 HU for women [2]. Although VAT area measurements provide additional context—with values >100 cm^2^ associated with metabolic syndrome and ≥160 cm^2^ considered “very high” risk—the density parameter demonstrates superior prognostic value for survival prediction in patients with transcatheter aortic valve implantation (TAVI) [12]. Both VAT cross-sectional area (cm^2^) and tissue density (Hounsfield Units) are measured, with density values utilized for metabolic risk assessment in this analysis. SAT is fat located beneath the skin; while generally less metabolically active than VAT, excessive amounts can still indicate obesity with significant mechanical and endocrine effects.

**Figure 1.**
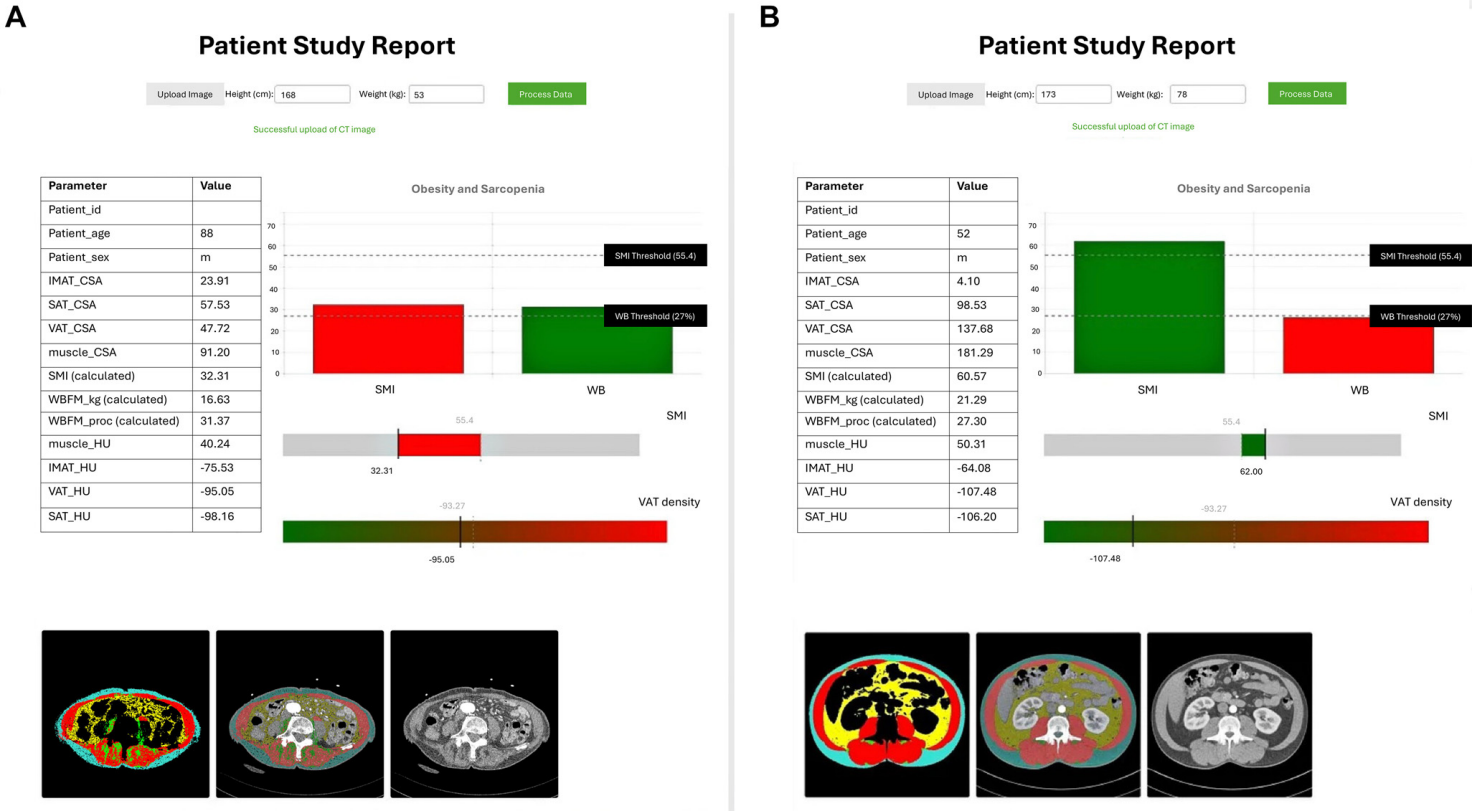
**Contrasting body composition phenotypes revealed through CT analysis.** Panel (A) presents a CT-derived body composition analysis of a patient with a low BMI (18.8 kg/m^2^) but significant sarcopenia. The analysis reveals a reduced skeletal muscle index (SMI 32.31), low muscle cross-sectional area (muscle_CSA 91.20), and elevated VAT density (−95.05). The color-coded CT segmentation displays muscle (red), visceral adipose tissue (yellow), and subcutaneous adipose tissue (blue) distribution, underscoring the limitations of BMI-based assessments. Although classified as “underweight” by BMI standards, this patient’s low muscle mass and unfavorable fat distribution indicate a significant metabolic risk. Panel (B) illustrates the CT analysis of a patient with a higher BMI (26.1 kg/m^2^) but preserved muscle mass. The analysis indicates a healthy skeletal muscle index (SMI 61.28), adequate muscle cross-sectional area (muscle_CSA 181.29), and lower VAT density (−107.48). The color-coded segmentation demonstrates a more favorable distribution of muscle and adipose tissue compartments. This example emphasizes that patients categorized as “overweight” by BMI may actually possess more advantageous body composition profiles and lower metabolic risk. These cases were selected from our clinical database as representative examples that illustrate contrasting body composition phenotypes, highlighting the limitations of BMI-based assessments. Both patients underwent clinically indicated CT imaging as part of their routine care. BMI: Body mass index; SMI: Skeletal muscle index; VAT: Visceral adipose tissue.

The efficacy of this approach is best illustrated through real-world examples. In our clinical experience, we have encountered numerous patients who highlight the limitations of BMI-based assessments (Figure 1). The first patient, with a BMI of 18.8 kg/m^2^, would traditionally be classified as underweight. However, CT analysis revealed an SMI of 32.31 cm^2^/m^2^, indicating significant sarcopenia, alongside concerning patterns of fat distribution. Conversely, a second patient, despite a BMI of 26.1 kg/m^2^—placing them in the overweight category—demonstrated preserved muscle mass with an SMI of 61.28 cm^2^/m^2^, reflecting a significantly healthier body composition profile.

### The role of artificial intelligence (AI)

The implementation of advanced body composition analysis has historically encountered significant barriers, including technical complexity, the necessity for specialized expertise, and considerable time investment. However, recent advancements in AI and deep learning have transformed this field, making intricate tissue analysis increasingly accessible for clinical application.

Fully automated algorithms are now capable of identifying the relevant CT slice and segmenting tissues within seconds, achieving accuracy comparable to expert manual analysis [13]. This advancement dramatically reduces analysis time to mere seconds while eliminating observer variability. Historically, manual segmentation of a single abdominal CT slice required 15–20 min of a technician’s time, rendering large-scale applications costly and time-consuming. Our experience indicates that AI automation has significantly enhanced efficiency, enabling the automatic reporting of body composition metrics for all abdominal CTs processed. The validated AI segmentation framework, AutoMATiCA, utilizes a U-Net neural network architecture and has been rigorously validated across 893 patients (80% training, 10% validation, 10% testing). In the testing cohort, dice similarity coefficient (DSC) scores exhibited excellent agreement between human and network-predicted segmentations, with processing speeds of approximately 350 milliseconds per scan on modern computing hardware [8].

It is crucial to highlight that these analyses typically utilize existing CT scans obtained for other clinical indications, rather than necessitating dedicated scans solely for body composition assessment. This “opportunistic” approach minimizes additional radiation exposure and costs, while maximizing the clinical value of imaging studies already performed [5, 14]. The increasing prevalence of CT imaging in routine clinical care—for oncologic staging, surveillance, cardiac evaluation, and various other indications—provides a wealth of imaging data that can be leveraged for body composition analysis without imposing additional patient risk or healthcare expenditure.

### Clinical applications across specialties

Evidence indicates that detailed CT-based tissue analysis is particularly beneficial in several clinical contexts by enhancing risk stratification and informing treatment decisions where BMI proves inadequate. Table 1 presents a comprehensive comparison of the limitations of BMI vs the advantages of CT-derived metrics across clinical specialties.

**Table 1:** Comparison of BMI and CT-derived body composition metrics across clinical specialties

**Clinical domain**	**BMI assessment limitations**	**CT-derived metrics advantages**	**Key study findings**	**Clinical implications**
General assessment	• Cannot distinguish between fat and muscle mass • No indication of fat distribution • Misclassifies muscular individuals as overweight	• Precise tissue quantification (SMI, VAT, SAT) • Distinction between different tissue types • Detailed fat distribution assessment	• Systematic review found CT analysis detected sarcopenia 27%–67% more frequently than BMI-based screening (Elhakim et al., 2023 [5])	• Improved phenotyping • Better risk stratification • More targeted interventions
Oncology	• Poor predictor of treatment toxicity • Limited prognostic value • Cannot identify sarcopenic obesity	• Identifies low muscle mass despite normal BMI • Quantifies metabolically active visceral fat • Predicts chemotherapy toxicity and surgical complications	• CT-derived metrics showed stronger prognostic value than BMI in breast cancer patients (Caan et al., 2018 [6]) • CT-based profiling improved outcomes prediction after oncologic liver surgery (Bernardi et al., 2022 [15])	• Better patient selection for therapy • Optimized chemotherapy dosing • Enhanced surgical risk assessment
Cardiology	• “Obesity paradox” confounds risk assessment • Unable to identify frail patients with normal BMI	• Identifies high-risk patients with sarcopenia • Detects visceral adiposity associated with cardiovascular risk	• CT-derived metrics predicted higher all-cause mortality in TAVI patients even when BMI was in “overweight” range (Pekař et al., 2024 [2])	• Refined risk stratification • Better patient selection for procedures • Improved post-procedural care planning
Surgery & critical care	• Poor predictor of post-surgical complications • Limited value in critical illness	• Predicts functional recovery • Identifies patients at risk for prolonged ventilation • Determines nutritional needs	• High visceral fat on CT more than doubled odds of postoperative complications in cancer surgery patients (van Helsdingen et al., 2024 [16]) • CT-measured visceral fat correlated with hospitalization risk in COVID-19 patients (Chandarana et al., 2021 [17])	• Tailored perioperative care • Targeted nutritional support • Enhanced rehabilitation planning
Clinical nutrition & frailty	• Cannot detect sarcopenic obesity • Misses muscle depletion in normal-weight patients	• Detects sarcopenia regardless of weight • Quantifies muscle quality (density) • Measures specific tissue compartments	• 63%–71% of normal-BMI hospitalized patients had muscle depletion detected on CT (Martin et al., 2024 [13])	• Personalized nutrition plans • Early intervention for sarcopenia • Targeted protein supplementation

SMI: Skeletal muscle index; VAT: Visceral adipose tissue; SAT: Subcutaneous adipose tissue; TAVI: Transcatheter aortic valve implantation; CT: Computed tomography; BMI: Body mass index. Statistical measures from cited studies: [5] Elhakim et al. (2023): This review found that CT body composition detects sarcopenia at rates 27.3%–66.7% higher than BMI-based detection methods across various clinical populations. [6] Caan et al. (2018): Among 3,241 nonmetastatic breast cancer patients, sarcopenia was linked to a 41% increase in mortality risk (HR ═ 1.41; 95% CI: 1.18–1.69), while high total adiposity was associated with a 35% increase in mortality risk (HR ═ 1.35; 95% CI: 1.08–1.69). Notably, BMI alone did not significantly correlate with overall mortality. [15] Bernardi et al. (2022): A comprehensive review of 33 studies involving 16,537 patients confirmed that CT body composition profiling is an established prognostic factor in hepatocellular carcinoma. Sarcopenia was consistently associated with poorer short- and long-term outcomes following liver surgery across multiple tumor types. [2] Pekař et al. (2024): In a cohort of 866 TAVI patients, AI-assisted CT analysis demonstrated that the skeletal muscle index (SMI HR ═ 0.986; 95% CI: 0.975–0.996), visceral adipose tissue density (VAT density HR ═ 1.015; 95% CI: 1.002–1.028), and subcutaneous adipose tissue density (SAT HR ═ 1.014; 95% CI: 1.004–1.023) significantly predicted all-cause mortality, with all *P* values < 0.05. [16] van Helsdingen et al. (2024): A meta-analysis of colorectal cancer surgery patients found that high visceral fat significantly increased the risk of overall postoperative complications (OR ═ 2.52; 95% CI: 1.58–4.00, *P* < 0.0001) and anastomotic leakage (OR ═ 1.76; 95% CI: 1.17–2.65, *P* ═ 0.006). [17] Chandarana et al. (2021): In COVID-19 patients, CT body composition analysis enhanced the prediction of hospitalization risk. The clinical model alone achieved an AUC of 0.70, while the inclusion of visceral adiposity improved the AUC to 0.73, and the optimal model incorporating muscle adiposity measures reached an AUC of 0.83. [13] Martin et al. (2024): A clinical implementation study indicated that registered dietitians utilizing CT skeletal muscle assessments identified muscle depletion in 63% of men (45/72) and 71% of women (17/24), with 94% of assessments completed in less than 15 minutes.

In oncology, patients exhibiting low skeletal muscle area on CT demonstrate significantly shorter survival and higher chemotherapy toxicity, independent of BMI. Bernardi et al. [15] found that CT-based body composition profiling improved outcome prediction following oncologic liver surgery. A meta-analysis by van Helsdingen et al. [16], involving over 16,500 cancer surgery patients, revealed that high visceral fat on CT more than doubled the odds of postoperative complications.

In cardiology, CT-derived metrics have refined risk assessment in ways that BMI cannot. Our own research, conducted by Pekař et al., [2] demonstrated that in patients undergoing TAVI, CT-derived metrics predicted higher all-cause mortality, despite the average BMI being classified as “overweight”. BMI alone proved misleading due to the obesity paradox in this elderly cohort, while CT measures successfully identified frail patients at high risk, who would otherwise appear “healthy” by BMI.

In surgical and critical care settings, we advocate for CT body composition analysis to provide greater granularity in risk stratification. CT-measured visceral fat correlates with hospitalization risk in COVID-19 patients, whereas BMI does not exhibit a similar relationship [17]. CT analysis reveals that muscle adiposity and visceral fat distribution provide crucial prognostic information independent of BMI. In critical illness, reduced muscle mass observed on imaging correlates with poorer outcomes, potentially identifying patients who may benefit from earlier physiotherapy or nutritional support.

A recent study by Chen et al. further demonstrated the clinical utility of CT-derived body composition metrics in patients with acute pancreatitis. Changes in SMI and pre-treatment skeletal muscle radiodensity were utilized to develop a metabolic score that accurately predicted disease severity, achieving AUC values of 0.764 and 0.741 in different patient populations [18]. This study exemplifies how CT body composition analysis can yield superior prognostic information compared to traditional clinical indicators across diverse acute care settings.

In clinical nutrition and frailty management, CT analysis aids in targeting therapy to those who genuinely require it. Martin et al. [13] implemented CT skeletal muscle assessments in clinical practice and discovered that 63%–71% of hospitalized patients with normal BMI actually exhibited muscle depletion detected on CT, leading to adjustments in protein and calorie provision. Through the early identification of sarcopenic obesity, clinicians can intervene before significant functional decline occurs, potentially altering disease trajectories. This enhanced diagnostic capability enables more precise risk stratification across various health outcomes, fundamentally transforming our approach to patient care. Moreover, the technology’s ability to accurately measure changes in body composition revolutionizes the design and monitoring of interventions, facilitating more targeted nutritional and exercise programs.

### Comparison with alternative methods

The spectrum of body composition assessment methods presents various advantages and limitations. Anthropometry (BMI, circumferences, skinfolds) is simple, inexpensive, and widely accessible; however, it lacks precision for individual assessments and fails to differentiate between fat and lean mass or evaluate fat distribution [3]. Bioelectrical impedance analysis (BIA) is portable, non-invasive, and yields quick results, but its accuracy is significantly influenced by hydration status and electrolyte balance, rendering it unreliable for patients with fluid imbalances [19]. Ackermans et al. (2022) noted that while BIA is relatively cost-effective and readily available, its accuracy diminishes in obese and cachectic patients due to the disproportion between body mass and conductivity. This limitation is particularly pertinent for patients with sarcopenic obesity, for whom CT-based measurements provide superior assessments of both muscle quantity and quality [20]. Dual-energy X-ray absorptiometry (DEXA) is regarded as a reference technique for body composition assessment, yet it has its shortcomings. Palmas et al. observed that although DEXA delivers accurate measurements of tissue mass, it does not differentiate between visceral and subcutaneous fat distribution. Their study validated that CT imaging, when analyzed with appropriate software and algorithms that account for both tissue area and density (Hounsfield Units), can achieve comparable accuracy to DEXA for body composition assessment in obese patients [19]. CT and MRI offer the most detailed insights into tissue distribution, albeit at a higher cost and complexity. They remain the gold standard for precise tissue quantification when such detail is clinically necessary [14]. While CT employs ionizing radiation, raising concerns regarding repeated scans, the context of opportunistic analyses mitigates this concern, as CTs are performed for legitimate medical reasons. Additionally, advancements in CT technology have significantly reduced radiation doses through low-dose techniques and iterative reconstruction algorithms. No extra radiation exposure occurs beyond that of the clinically indicated scan, and if a CT were conducted primarily for body composition analysis, it could be performed at a low dose, focusing on the L3 region.

This context elucidates why BMI continues to be utilized, primarily due to its simplicity and established reference ranges, while advanced imaging techniques provide substantial clinical benefits. Bazzocchi et al. [14] emphasized that although CT and MRI are highly accurate, their use has been constrained by cost and accessibility; however, automation and increased imaging frequency are beginning to overcome these barriers.

### Implementation considerations

#### Challenges and solutions

The implementation of CT-derived body composition analysis encounters several interconnected challenges that necessitate practical solutions. Concerns regarding radiation exposure naturally arise in discussions of CT-based assessments. Nevertheless, within the framework of opportunistic analysis, these concerns are significantly alleviated since the CT is performed for valid medical reasons, with modern technology effectively reducing radiation doses through low-dose techniques and iterative reconstruction algorithms [5]. Ackermans et al. (2022) [20] underscore that the “opportunistic” use of existing CT scans is a pragmatic approach to address these concerns, facilitating muscle analysis without additional radiation exposure to the patient.

Cost and accessibility represent significant challenges as well. Although operating CT scanners incurs high expenses, AI-powered analysis dramatically enhances accessibility by automating measurements that previously required specialized expertise [14]. The incremental cost of deriving body composition from existing CT scans is minimal with automation, especially when juxtaposed with the potential clinical value of identifying high-risk individuals. This transformation redefines the cost-benefit equation by adding substantial diagnostic value to imaging studies already performed.

Integrating this technology into existing workflows poses practical obstacles that must not be overlooked. Martin et al. [13] found that when introducing CT muscle measurement in clinical nutrition practice, the primary barriers were cumbersome processes for image acquisition and the integration of analysis software into existing systems. However, once established, clinicians reported that these measurements positively contributed to their nutrition care practices.

In our view, the most pressing need is to establish robust reference values across diverse populations and to standardize these new systems within existing clinical workflows. As AI platforms continue to evolve, we anticipate increasingly sophisticated risk assessments, enhanced accessibility through cloud-based processing, and greater integration with other imaging modalities, fostering a more comprehensive approach to body composition assessment.

### Acknowledgment of methodological limitations

While we advocate for the clinical adoption of CT-based body composition analysis, it is crucial to recognize several methodological limitations. Current research, although promising, is heterogeneous in analytical approaches, complicating direct comparisons between studies. The field lacks standardized cutoff values across diverse populations, particularly among ethnic minorities, pediatric patients, and individuals with specific disease states. Technical variability among different CT scanner protocols, contrast phases, and reconstruction algorithms may affect tissue attenuation values and segmentation accuracy. Implementation studies have primarily been conducted in academic medical centers with specialized expertise, which may limit generalizability to community settings. Furthermore, while AI automation has significantly enhanced efficiency, the opaque nature of some algorithms raises concerns regarding interpretability and regulatory oversight. Longitudinal validation studies that investigate how these metrics evolve over time and with interventions are still scarce. A transparent acknowledgment of these limitations not only underscores the need for methodological refinement but also emphasizes the ongoing research required to establish this promising technology as a clinical standard.

### Practical implementation strategy

For institutions aiming to integrate AI-powered CT body composition analysis, our recent experience with a web-based interface for TAVI patient assessment provides valuable insights. We developed a user-friendly system that combines AutoMATiCA’s validated AI segmentation capabilities [8] with an intuitive clinical interface, reducing analysis time to approximately 21 s from image upload to results display.

The ESPEN-EASO diagnostic algorithm recommends a two-stage approach: initially screening high-risk individuals using BMI or waist circumference alongside clinical risk factors, followed by a comprehensive assessment of muscle function and body composition, which aligns with our proposed AI-powered workflow [4].

The implementation operates on standard hospital infrastructure, requiring minimal technical expertise from clinicians while offering comprehensive visualization of body composition metrics, including SMI, VAT, and SAT. User experience validation with clinicians from various specialties confirmed that the most valued features were clear visual representations of obesity and sarcopenia metrics, as well as immediate access to clinical implications.

Our case studies illustrated the system’s capacity to identify critical conditions such as sarcopenic obesity, which would be overlooked by BMI assessment alone, thereby providing compelling evidence for clinical adoption (Fig. 1). The key to successful implementation lies not in technical sophistication but in seamless workflow integration that converts complex analytical capabilities into actionable insights without disrupting established clinical processes.

We recommend a staged implementation approach for institutions interested in adopting this technology. Institutions should begin with opportunistic analyses of existing CT scans for high-risk patient groups, where the clinical impact would be most immediate. Subsequently, they should establish local reference values based on their specific patient populations to ensure appropriate contextual interpretation. Integration of reporting into standard radiology workflows is essential for sustainability, followed by the development of clinical decision pathways that incorporate body composition metrics. Finally, targeted education for clinicians on interpretation and clinical applications will ensure optimal utilization of these new metrics in daily practice.

### Future directions

We anticipate several promising developments in CT-based body composition analysis that will further enhance its clinical utility. The incorporation of AI beyond simple segmentation towards predictive modeling represents a significant frontier. By integrating body composition metrics with other clinical variables (e.g., laboratory values, functional assessments, and comorbidities), machine learning algorithms could generate personalized risk profiles and treatment recommendations. Continued refinement of population-specific reference values across ethnic groups, age ranges, and disease states is expected to address critical gaps in current implementation. Multi-center validation studies and consensus initiatives led by professional societies will likely establish standardized reporting frameworks and integration pathways. Additionally, the development of cloud-based processing platforms could democratize access to advanced analytics, making them available even in resource-limited settings. Future research should focus on demonstrating how these imaging biomarkers can guide personalized interventions that meaningfully improve clinical outcomes, moving beyond mere risk stratification to directly inform therapeutic decisions across medical specialties.

## Conclusion

While BMI remains valuable for population-level screening, evidence increasingly suggests that optimal individual patient care necessitates more sophisticated analyses than BMI alone can provide. The evidence reviewed in this paper demonstrates that CT-based metrics often outperform BMI in predicting significant clinical outcomes across multiple specialties, including oncology, cardiology, and critical care.

The opportunistic approach of extracting body composition data from clinically indicated CT scans addresses both radiation exposure and cost concerns. By leveraging AI automation, what was once a labor-intensive process requiring specialized expertise has become increasingly accessible for routine clinical implementation.

We assert that the future of body composition assessment lies in embracing these more sophisticated approaches that acknowledge the limitations of traditional anthropometrics. As we work to standardize these techniques and integrate them into clinical workflows, we move closer to our goal of providing truly personalized patient care based on an objective, detailed understanding of individual body composition. This shift represents not merely a technical advancement but a significant improvement in how we assess, stratify, and treat patients across the spectrum of medical specialties.

**Declaration of generative AI in scientific writing:** During the preparation of this work, the authors used Claude 3.7 Sonnet to assist with the writing process. After using this tool, the authors reviewed and edited the content as needed and take full responsibility for the content of the publication.

**Ethical statement:** This opinion paper includes de-identified patient CT images and body composition data (Figure 1) from cases that received ethical approval from the Ethics Committee of Hospital AGEL Trinec-Podlesi (approval number: EK 301/22). No patient-identifiable data is included in the presented examples.
